# A Subunit Vaccine Harboring the Fusion Capsid Proteins of Porcine Circovirus Types 2, 3, and 4 Induces Protective Immune Responses in a Mouse Model

**DOI:** 10.3390/v16121964

**Published:** 2024-12-23

**Authors:** Qikai Wang, Ran Zhang, Yue Wang, Ying Wang, Libin Liang, Haili Ma, Haidong Wang, Longlong Si, Xingchen Wu

**Affiliations:** 1 College of Veterinary Medicine, Shanxi Agricultural University, Jinzhong 030801, China; z20213668@stu.sxau.edu.cn (Q.W.);; 2 Key Laboratory of Quantitative Synthetic Biology, Shenzhen Institute of Synthetic Biology, Shenzhen Institute of Advanced Technology, Chinese Academy of Sciences, Shenzhen 518055, China; 3 College of Life Sciences, University of Chinese Academy of Sciences, Beijing 100049, China; 4 Department of Medicine, Emory University, Atlanta, GA 30322, USA

**Keywords:** porcine circovirus, capsid, antigenic epitope, fusion protein, subunit vaccine, immunogenicity

## Abstract

Coinfections with porcine circovirus types 2, 3, and 4 (PCV2, PCV3, and PCV4) are increasingly being detected in the swine industry. However, there is no commercially available vaccine which prevents coinfection with PCV2, PCV3, and PCV4. The development of a vaccine expressing capsid (Cap) fusion proteins of multiple PCVs represents a promising approach for broadly preventing infection with PCVs. In this study, we developed a PCV subunit vaccine candidate (Cap 2-3-4) by predicting, screening, and fusing antigenic epitopes of Cap proteins of PCV2, PCV3, and PCV4. Immunoprotection assays showed that the prokaryotic expression of Cap 2-3-4 could effectively induce high levels of PCV2, PCV3, and PCV4 Cap-specific antibodies and successfully neutralize both PCV2 and PCV3. Furthermore, Cap 2-3-4 demonstrated a potent ability to activate cellular immunity and thus prevent lung damage in mice. This study provides a new option for the development of broad vaccines against PCVs.

## 1. Introduction

Porcine circovirus (PCV) belongs to the genus *Circovirus* within the family *Circoviridae* [1]. PCV2 infection is known to lead to immunosuppression, reducing resistance to various pathogens and causing secondary infections [2,3,4]. The clinical symptoms of PCV3 infection are similar to those of porcine dermatitis and nephropathy syndrome [5]. The detection of PCV3 in sow colostrum has highlighted the possibility of early transmission [6]. PCV4 infection has been proven to induce reproductive dysfunction, resulting in the delivery of dead or mummified fetuses, as well as diarrhea and other symptoms [7,8,9]. However, there are no commercially available vaccines for PCV3 and PCV4.

The genome of PCV2 is approximately 1767 bp in length; it contains 11 open reading frames (ORFs), with ORF2 encoding the structural protein Cap, which is the only structural protein of PCV2 [10,11]. The genome of PCV3 is about 2000 bp in length. Genetic evolution analysis has indicated that PCV3 shares approximately 37% of its homologous sequences with PCV2 [12,13]. PCV3 consists primarily of three ORFs, with ORF2 again encoding the unique structural protein Cap. The genome of PCV4 is approximately 1770 bp in length, and the functions of its proteins encoded by ORFs are similar to those of the proteins encoded by the ORFs of PCV2 and PCV3 [14]. As a major immunogen, Cap is the only structural protein of PCV, and it contains antigenic epitopes which induce immune response [15,16]. The antigenic epitopes of PCV2 Cap can bind to surface receptors of lymphocytes, activate immune responses, and stimulate the body to produce specific neutralizing antibodies. In addition, Cap can elicit cellular immune response against infection. Researchers engaged in developing genetically engineered vaccines against PCV2 have mainly focused on Cap as a research hotspot [17]. A better understanding of the antigenic epitopes of PCV2 Cap (Cap2), PCV3 Cap (Cap3), and PCV4 Cap (Cap4) is crucial for the development of effective vaccines against coinfections involving these viruses. As an important type of vaccine, the subunit vaccine involves introducing protective antigen genes from pathogenic microorganisms into host systems, such as bacteria, yeast, or animal cells, to perform high-efficiency expression [18].

The first step in preparing protein subunit vaccines is to identify and isolate protein subunits from the pathogen; next, a genomic plasmid expressing the viral protective antigens is constructed for the expression and purification of antigens. The purified protein is then conjugated with an adjuvant to prepare vaccines. Compared with traditional inactivated and attenuated vaccines, subunit vaccines offer several advantages, including superior safety profiles, the ability to trigger a strong and effective immune response without causing disease, high production efficiency, and easy storage and transportation [19]. Major systems, including the expression systems of *E. coli*, baculovirus, yeast, and mammals, play pivotal roles in the efficient expression of protein subunits [20]. In particular, the *E. coli* expression system is characterized by rapid production and efficient expression. However, this system cannot post-translationally modify eukaryotic proteins. Importantly, proteins lacking envelope and glycosylation sites can be effectively expressed in the *E. coli* system [21]. Although the baculovirus expression system enables the post-translational modification and processing of eukaryotic proteins during expression, this system is generally more costly than the *E. coli* expression system.

Cap is the only structural protein of PCV, and it contains antigenic epitopes [15]. PCV Cap antigenic epitopes can bind to surface receptors of lymphocytes, activating immune responses. The identification and screening of antigenic epitopes can be seen as pivotal in the development of subunit vaccines, immune diagnostic tests, and antibody production. Currently, three subunit vaccines, CircoflexTM, Circumvent, and Porcilis PCV, are approved for use on the international market. In one study of piglets immunized with Porcilis PCV, it was found that production of PCV2-specific neutralizing antibodies was triggered, preventing the development of PCV2 viremia [22]. Importantly, previous studies have demonstrated that mice might serve as an animal model for porcine circovirus infection, enabling an elucidation of pathogenesis following viral infection and facilitating the evaluation of antiviral drugs and candidate vaccines [23,24,25,26,27]. However, no PCV3 or PCV4 vaccines are yet commercially available. In this study, we used online software to perform bioinformatics analysis on Cap2, Cap3, and Cap4. From an analysis of the B-cell epitopes of the Cap proteins, we designed three multi-epitope fusion expression vectors based on the dominant antigenic epitopes of Cap proteins, namely LCap 2-3-4, with a longer sequence; SCap 2-3-4, with a shorter sequence; and ECap 2-3-4, with a truncated Cap which lacked the nuclear localization signal (NLS). These fusion proteins were produced by prokaryotic and baculovirus expression systems so that optimal expression conditions could be explored. Moreover, we identified the optimal fusion antigenic epitopes by testing the immunization effects of different fusion proteins, with the aim of developing a more economical epitope peptide vaccine. These findings are expected to provide a scientific foundation for developing a novel fusion vaccine against PCV2, PCV3, and PCV4.

## 2. Materials and Methods

### 2.1. Viruses and Cells

The genomic sequences of isolated PCV2 and PCV3 strains were uploaded to the National Centre for Biotechnology Information (NCBI) database (GenBank accession numbers PP566975 and PP566974, respectively). The genomic sequence of PCV4 was obtained from a published strain (GenBank accession number MW600958.1). PCV2 and PCV3 strains were stored in our laboratory and propagated in porcine kidney 15 cells (PK-15). Viral copies were measured using quantitative polymerase chain reaction (qPCR). The SF9 (*Spodoptera frugiperda*) and PK-15 cell lines were stocked in the Microbiology and Immunology Laboratory of Shanxi Agricultural University. Spodoptera frugiperda cells (SF9) were cultured with SIM-SF medium (Sino Biological, Beijing, China), and PK-15 cells were cultured in high-glucose Dulbecco’s modified essential medium (DMEM) (Invitrogen, Carlsbad, CA, USA) supplemented with 10% heat-inactivated fetal bovine serum (Tianhang Biotechnology, Huzhou, China), 100 U/mL penicillin, and 0.1 mg/mL streptomycin. All cells (PK-15 and SF9) were used at the exponential growth phase.

### 2.2. Screening of Antigenic Epitopes in the Cap Proteins of PCV2, PCV3, and PCV4

Prot Param (https://web.expasy.org/protparam/) (accessed on 17 March 2024) software was used to analyze the molecular formulas, amino acid composition, and isoelectric points of Cap2, Cap3, and Cap4. Signal 5.0 (SignalP 5.0-DTU Health Tech-Bioinformatic Services) software was used to predict the signal peptides of Cap proteins. DNA Star 7.1 software was used to analyze the α-helices, hydrophobicity, flexible regions, and B-cell epitopes of Cap proteins. The Garnier–Robson and Chou–Fasman methods were used to predict the α-helices of Cap proteins. The Kyte–Doolittle method was used to analyze the hydrophobicity of Cap proteins. The Karplus–Schulz method was used to analyze the flexible regions of Cap proteins. The Jameson–Wolf method was used to analyze the detected B-cell antigenic epitopes of Cap proteins. The IEDB Analysis Resource (http://tools.iedb.org/bcell/result/) (accessed on 17 March 2024) and BCPRE SERVER (http://ailab-projects2.ist.psu.edu/bcpred/index.html) (accessed on 17 March 2024) software packages were used to analyze the predicted B-cell antigenic epitopes of Cap proteins.

### 2.3. Plasmid Construction

**Figure 1:**
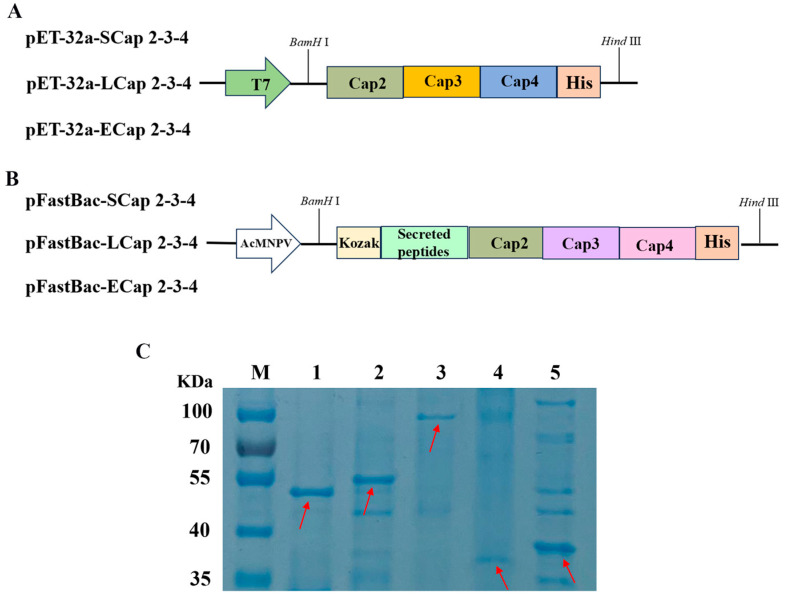
Plasmid construction process and protein purification. (**A**,**B**) Schematic diagram of vector construction. T7 and AcMNPV represent the promoters in the vectors. SCap 2-3-4 represents a combination of shorter predicted B-cell epitopes from Cap2, Cap3, and Cap4. LCap 2-3-4 is a combination of longer B-cell epitopes. ECap 2-3-4 is a combination of Cap2, Cap3, and Cap4 without NLS. (**C**) SDS–PAGE results for purified proteins. M: protein marker; 1: pSCap 2-3-4; 2: pLCap 2-3-4; 3: pECap 2-3-4; 4: rSCap 2-3-4; 5: rLCap2-3-4. The positions of purified protein were indicated by the location marked with the red arrow.

Cap2, Cap3, and Cap4 were codon-optimized on the preference of *E. coli* codons. In this study, the antigenic epitopes predicted from PCV2, PCV3, and PCV4 Cap proteins were linked using Linker (GGGGS). All different Cap proteins were linked to each other using Linker (GSGGGGGSGGGGSGS). The His-tag sequence (HHHHHH) was inserted into the C-termini of different combination sequences of Cap 2-3-4 and then ligated into the pet-32a vector (GENEWIZ Biotechnology, Suzhou, China) (Figure 1A). A sequence lacking the optimized NLS at the N-termini of Cap2, Cap3, and Cap4 was used to construct recombinant plasmid pET-32a-ECap 2-3-4. For the baculovirus–insect cell expression system, the Kozak sequence (GCCACC) and a secreted peptide (MHSSALLCCLVLLTGVRA) were inserted into the N-terminus of the sequence. A His-tag sequence was inserted into the C-termini of various Cap 2-3-4 sequence combinations. Furthermore, a linker (GGGGS) was used to connect the predicted antigenic epitopes of Cap2, Cap3, and Cap4, while a linker (GSGGGGGSGGGGGSGS) was used between the different Cap proteins; the designed sequences were then inserted into a pFastBac1 vector to obtain recombinant plasmid pFastBac-SCap 2-3-4, pFastBac-LCap 2-3-4, and pFastBac-ECap 2-3-4 (Figure 1B). All the sequences were synthesized using Sangon biotechnology and cloned into vectors (Figure S4).

### 2.4. Protein Expression and Purification

The Rosetta strain was used to express the recombinant plasmids pET32a-SCap 2-3-4, pET32a-LCap 2-3-4, and pET-32a-ECap 2-3-4, resulting in production of their respective proteins. Similarly, the SF9 cells expressed rSCap 2-3-4, rLCap 2-3-4, and rECap 2-3-4 proteins, followed by pFastBac-SCap 2-3-4, pFastBac-LCap 2-3-4, and pFastBac-ECap 2-3-4 transfection. These supernatants containing proteins (pSCap 2-3-4, pLCap2-3-4, pECap 2-3-4, rSCap 2-3-4, and rLCap 2-3-4) were purified through a HisTrap HP 5 mL column (Cytiva, MA, USA); the target proteins were then rinsed using a buffer consisting of 20 mM Tris (pH 8.0), 150 mM NaCl, and 300 mM imidazole.

### 2.5. Animal Experiment

Seven-week-old BALB/c mice were randomly assigned to 8 groups, with 15 mice per group. One group acted as a control (Mock), while the remaining 7 groups were immunized with phosphate-buffered saline (PBS), Cap 2-3-4 (100 μg), and the commercial PCV2 vaccine (100 μg) (YEBIO, Qingdao, China) through intramuscular injection at 0 day (d) and 14 d. Cap 2-3-4 was mixed with Freund’s adjuvant (Merck, MA, USA) for injection. The commercial PCV2 vaccine (manufactured by YEBIO, Qingdao, China) is a subunit vaccine derived from the Cap protein of PCV2b, formulated into a suspension with an adjuvant for sale. The Cap-specific antibody levels were detected weekly post-vaccination. Serum was collected at 28 d and 35 d after first vaccination to measure virus-neutralizing antibody levels. At 28 d and 35 d post-vaccination, 5 mice from each group were euthanized for splenic lymphocyte proliferation assays and enzyme-linked immunosorbent assays (ELISAs) to detect IL-4 and IFN-γ levels. At 35 d post-vaccination, all mice were challenged with an intraperitoneal injection of 1 × 10^5^ copies of PCV2 and PCV3, except those in the Mock group. These mice were euthanized at 63 d post-vaccination for tissue collection.

### 2.6. Enzyme-Linked Immunosorbent Assay

Serum samples were collected from 5 mice selected randomly from each group at 7, 14, 21, 28, and 35 d post-vaccination and subjected to the detection of IgG antibodies against PCV2 and PCV3 using PCV2 and PCV3 antibody detection kits (Sennoside Biotechnology, Chuzhou, China), according to the manufacturer’s instructions. Levels of PCV4-specific IgG antibodies in the serum were measured using an ELISA. Recombinant PCV4-Cap proteins were diluted to 20 ng/µL in ELISA coating buffer (Solarbio, Beijing, China). Amounts of 100 µL of diluted solution were added to the wells of a 96-well ELISA plate and incubated overnight at 4 °C. After removing the PCV4-Cap protein solution, washing was carried out 3 times with washing buffer (PBS containing 0.1% Tween 20); 100 μL amounts of blocking buffer (1% Bovine Serum Albumin, BSA) were then added to each well and incubated for 1 h at 37 °C. After discarding the blocking buffer and carrying out further washing, 100 µL amounts of diluted serum samples were added to each well and incubated for 2 h at 37 °C. Following 3 additional washes, 100 µL amounts of HRP-conjugated anti-mouse IgG (Proteintech, Wuhan, China) diluted in blocking buffer were added for 1.5 h at 37 °C. After five washes, 100 µL amounts of Tetramethylbenzidine (TMB) substrate (Solarbio) were used for detection, and the reaction was halted after 15 min using 100 µL of ELISA stop solution (Solarbio). The optical density of the samples at 450 nm was detected using a microplate reader. For the detection of IL-4 and IFN-γ (MEIMIAN, Jiangsu, China), cell supernatants were measured according to the manufacturer’s guidelines using a commercial ELISA kit.

### 2.7. Virus Neutralization Test

Starting with a 1:2 ratio, the serum was serially diluted with culture medium. Subsequently, 50 μL diluted serum was mixed with 50 μL 200 TCID50/100 μL of PCV2 or PCV3 and then incubated at 37 °C for 1 h. The mixture was then added to a 96-well plate containing PK-15 cells for infection. After 72 h, the cells were incubated with rabbit anti-Cap serum at 4 °C and labeled with FITC-conjugated goat anti-rabbit IgG [28].

### 2.8. Spleen Lymphocyte Proliferation Assay

Following the manufacturer’s instructions, spleen lymphocytes were isolated using lymphocyte isolation medium (Solarbio). The isolated splenic lymphocytes were seeded into a 96-well plate and stimulated with 1 multiplicity of infection (MOI) of PCV2 or PCV3, 5 μg/mL of Concanavalin A (ConA), or culture medium for 48 h. Then, 20 μL CCK-8 solution was added to each well in the dark, followed by incubation for 2 h. The absorbance was measured at a wavelength of 450 nm. The result was calculated using the following formula: (OD value of the PCV3-stimulated well—OD of the blank well) divided by (OD of the negative control well—OD of the blank well).

### 2.9. Pathological Histology

After 28 d of challenging, the lungs of mice were harvested and fixed in 4% paraformaldehyde. The prepared paraffin sections were sectioned using a HistoCore fully automated slicer (Leica Biosystems, Wetzlar, Germany). Lung lesions of mice were then observed under a microscope after hematoxylin and eosin (HE) staining.

### 2.10. Statistical Analysis

All statistical analyses were performed using GraphPad Prism 9 (GraphPad Inc., San Diego, CA, USA). All data were presented in the form of mean ± SD. A one-way analysis of variance (ANOVA) was conducted to assess multiple groups. *p* values for multiple comparisons were then obtained, and Bonferroni post hoc testing was carried out. Statistical significance was defined as *p* < 0.05.

## 3. Results

### 3.1. Prediction and Screening of Antigenic Epitopes in Cap Proteins

To predict antigenic epitopes of Cap proteins, the physicochemical properties, signal peptides, transmembrane domains, and secondary structures of Cap proteins were analyzed using online software packages such as Prot param, Signal 5.0, TMHMM, and DNA star. The results indicate that Cap proteins possessed stable physicochemical properties and belonged to the group of hydrophilic proteins (Table S1). At the same time, Cap2, Cap3, and Cap4 were identified as non-transmembrane proteins without signal peptides in their sequences (Figure S1). An analysis of structure demonstrated that the Cap protein contained a lower abundance of α-helices and a higher prevalence of linear regions. Regions with these structural features are more likely to interact with antibodies. In addition, the Cap proteins exhibited numerous flexible regions, which contributed to the formation of antigenic epitopes (Figure S2). Additionally, the B-cell epitopes of Cap proteins were predicted with BCPRE SERVER and IEDB software. Based on the antigenic epitope score prediction results, we designed three groups of antigenic epitopes for combinatorial tandem linkage (Figure S3), namely SCap 2-3-4, with fewer epitopes; LCap 2-3-4, with more epitopes; and ECap 2-3-4, with a full-length Cap lacking an NLS (Table 1).

### 3.2. Plasmid Construction and Protein Purification

To obtain an effective subunit vaccine against PCV2, PCV3, and PCV4, we constructed a fusion expression plasmid with indicated antigenic epitopes of Cap. Based on the preference of *E. coli*, Cap2, Cap3, and Cap4 codon sequences were optimized to engineer pET32a-SCap 2-3-4, pET32a-LCap 2-3-4, and pET32a-ECap 2-3-4 plasmids (Figure 1A). To further enhance protein expression in the baculovirus–insect cell expression system, we optimized codons of Cap2, Cap3, and Cap4 and strategically inserted Kozak and secretory peptide sequences at the 5′ end, generating pFastBac-SCap 2-3-4, pFastBac-LCap 2-3-4, and pFastBac-ECap 2-3-4 plasmids (Figure 1B). The proteins expressed by pET32a-SCap 2-3-4, pET32a-LCap 2-3-4, pET32a-ECap 2-3-4, pFastBac-SCap 2-3-4, pFastBac-LCap 2-3-4, and pFastBac-ECap 2-3-4 were represented by pSCap 2-3-4, pLCap 2-3-4, pECap 2-3-4, rSCap 2-3-4, rLCap 2-3-4, and rECap 2-3-4, respectively. However, rECap 2-3-4 could not be expressed. The purified pSCap2-3, pLCap2-3, pECap2-3, rSCap2-3, and rLCap2-3 were analyzed using SDS-PAGE, and the protein sizes were found to be approximately 46 KDa, 58 KDa, 88 KDa, 34 KDa, and 38 KDa, respectively (Figure 1C).

### 3.3. pSCap 2-3-4 Activated Higher Humoral Immune Responses than the Other Inoculated Groups

**Figure 2:**
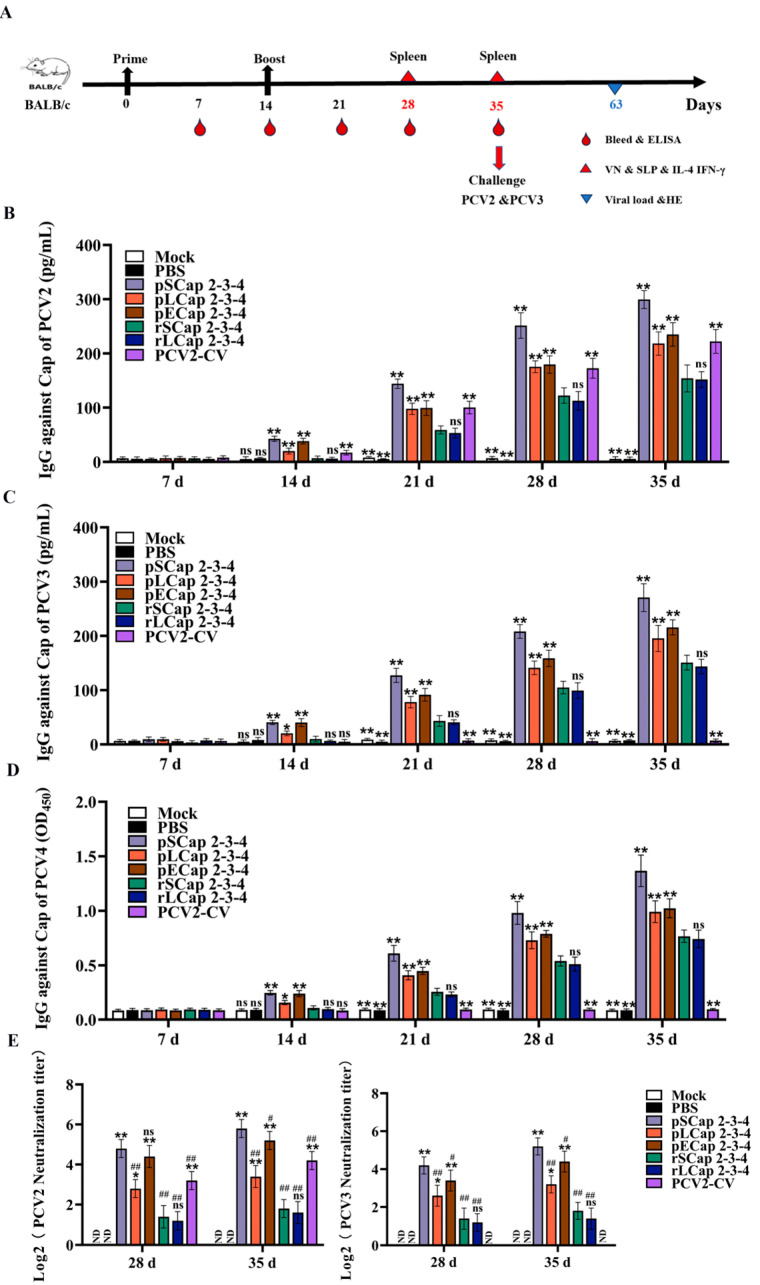
Immunogenicity evaluation of recombinant protein vaccines. (**A**) The mouse immunization and challenge experimental process. (**B**–**D**) The levels of specific antibodies against Cap2, Cap3, and Cap4 in the serum were measured at the indicated times using ELISAs. (**E**) The harvested serum samples at 28 d and 35 d post-inoculation were incubated with PCV2 and PCV3, respectively. PCV2- and PCV3-neutralizing antibody levels in PK-15 cells were analyzed after stimulation with the treated serum for 72 h. ND indicates no detection. * *p* < 0.05, ** *p* < 0.01 versus rSCap 2-3-4 immunized group at the same time point (*n* = 5); ^#^ *p* < 0.05, ^##^ *p* < 0.01 versus pSCap 2-3-4 immunized group at the same time point (*n* = 5); ns: no significant difference.

To assess the humoral immunogenicity of fusion proteins, serum antibody titers were monitored at 7 d intervals after vaccination at 0 d and 14 d using various fusion proteins and a commercial PCV2 vaccine (Figure 2A). The results demonstrate that the special antibody titers of PCV2, PCV3, and PCV4 increased significantly following booster vaccination. Interestingly, at 21 d, 28 d, and 35 d post-inoculation, the concentrations of anti-PCV2 antibodies in the pSCap 2-3-4, pLCap 2-3-4, pECap 2-3-4, and PCV2-CV groups were significantly higher than those in the rSCap 2-3-4 and rLCap 2-3-4 groups (Figure 2B). Anti-PCV3 and anti-PCV4 antibodies were not detected in the PCV2-CV group (Figure 2C,D). In addition, the levels of neutralizing antibodies against both PCV2 and PCV3 in the pSCap 2-3-4 and pECap 2-3-4 groups were obviously higher compared with other groups, and pSCap 2-3-4 achieved the highest neutralizing antibody levels at 35 d post-inoculation (Figure 2E). These findings indicate that the pSCap 2-3-4 vaccine can stimulate a strong humoral immune response, effectively neutralizing both PCV2 and PCV3 in mice.

### 3.4. pSCap 2-3-4 Activated Higher Cellular Immune Responses than the Other Inoculation Groups

**Figure 3:**
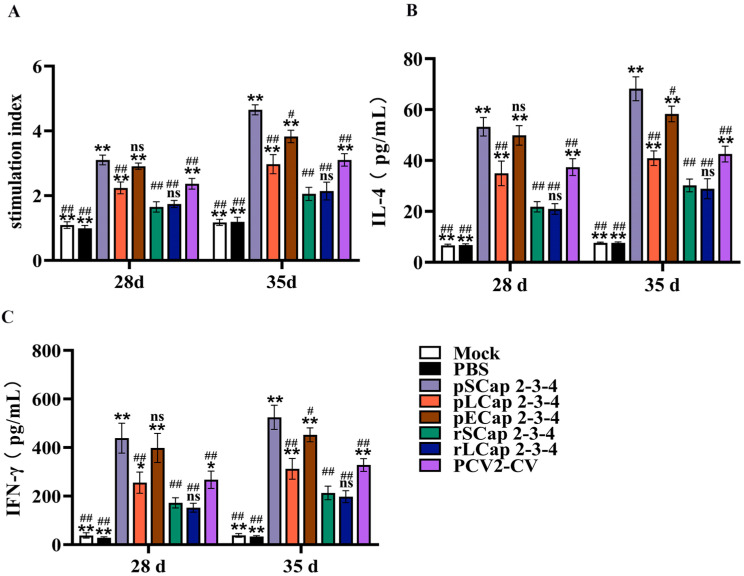
Detection of splenic lymphocyte proliferation and cytokine levels. (**A**) The isolated splenic lymphocytes were further stimulated with PCV2 and PCV3 for 48 h following ConA; this served as a positive control while the medium served as a negative control. A CCK-8 assay was employed to detect lymphocyte proliferation. (**B**,**C**) The levels of IL-4 and IFN-γ in lymphocyte supernatants were measured from different groups after challenging with PCV2 and PCV3. * *p* < 0.05, ** *p* < 0.01 versus rSCap 2-3-4 immunized group at the same time point (*n* = 5); ^#^ *p* < 0.05, ^##^ *p* < 0.01 versus pSCap 2-3-4 immunized group at the same time point (*n* = 5); ns: no significant difference.

To evaluate the elicited cellular immune response elicited in mice, splenic lymphocytes were isolated at 28 d and 35 d post-inoculation to measure proliferative capacity. The results showed that lymphocyte proliferation in the pSCap 2-3-4, pLCap 2-3-4, pECap 2-3-4, and PCV2-CV groups was higher than in the rSCap 2-3-4 and rLCap 2-3-4 groups at 35 d post-inoculation. Notably, the pSCap 2-3-4 group exhibited significantly higher proliferation rates than the pLCap 2-3-4, pECap 2-3-4, and PCV2-CV groups (Figure 3A). Furthermore, the pSCap 2-3-4, pLCap 2-3-4, pECap 2-3-4, and PCV2-CV treatments produced higher levels of IL-4 and IFN-γ than rSCap 2-3-4 and rLCap 2-3-4 (Figure 3B,C), and the levels of IL-4 and IFN-γ in the pSCap 2-3-4 group were significantly higher than those in the pLCap 2-3-4, pECap 2-3-4, and PCV2-CV groups at 35 d post-inoculation. These findings underscore the potent capacity of pSCap 2-3-4 to activate cellular immune responses in mice.

### 3.5. The Viral Load Was Reduced and Lung Pathology Was Less Severe in Mice Vaccinated with pSCap 2-3-4 Compared to Other Groups

**Figure 4:**
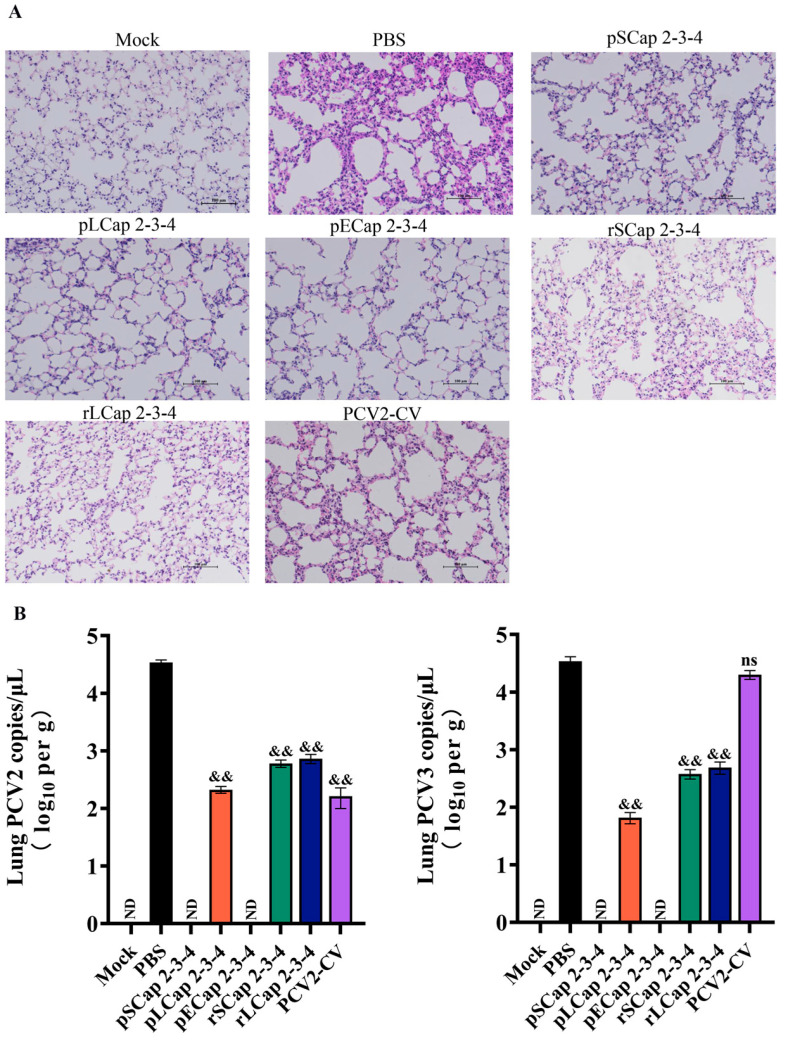
Detection of pathological changes in mouse lung tissue and virus copy numbers. (**A**) These lungs were processed and embedded in paraffin and sectioned for histological staining. Lung sections were stained with HE and examined microscopically to observe pathological changes, with a 100 μm scale bar. (**B**) The copies of PCV2 and PCV3 in lungs from each group were quantified using qPCR. ND indicates no detection. ^&&^ *p* < 0.01 versus PBS immunized group at the same time point (*n* = 5); ns: no significant difference.

To evaluate the protective efficacy of vaccines against PCV2 and PCV3 infections, we conducted a histological analysis of lung tissues from mice challenged with these viruses for 28 d. Our results showed that the PBS group exhibited significant pathological lesions, including inflammatory cell infiltration and alveolar wall thickening. The rLCap 2-3-4 and rSCap 2-3-4 groups showed moderate lung lesions, although less severe than those in the PBS group. Importantly, the pLCap 2-3-4, pSCap 2-3-4, and pECap 2-3-4 immunized groups of mice exhibited almost no significant lesions. However, the PCV2 vaccine group exhibited significant alveolar wall thickening and increased inflammatory factors, indicating that the PCV2 vaccine did not play a good protective role (Figure 4A). In addition, viral loads in lungs were detected at 28 d post-infection. Our results indicate that PCV2 viral loads were significantly reduced in all immunized groups compared with the PBS group, with the pSCap 2-3-4 group showing the lowest levels among the vaccinated groups (Figure 4B). Levels of pCV3 viral load were lower in pSCap 2-3-4, pLCap 2-3-4, pECap 2-3-4, rSCap 2-3-4, and rLCap 2-3-4 groups than in the PBS group, with the lowest levels being recorded in the pSCap 2-3-4 group. There was no difference between the PCV2-CV group and the PBS group in terms of PCV3 viral loads (Figure 4B). These findings highlight the efficacy of the pSCap2-3-4 vaccine in inhibiting viral replication and alleviating lung pathology following PCV2 and PCV3 challenges.

## 4. Discussion

PCV2, PCV3, and PCV4 have become widely prevalent globally, and coinfections with these viruses are increasingly being detected in the swine industry [29,30,31]. PCV2, PCV3, and PCV4 infections exhibit similar clinical symptoms, posing challenges for diagnosis and prevention [32]. Vaccination remains the most effective strategy for preventing and controlling PCV infections. Cap is the only structural protein of PCV; it contains numerous antigenic epitopes that determine its antigenic epitopes by binding to surface receptors on lymphocytes, thus activating the immune response. Previous studies have shown that the fusion expression of antigens and their epitopes can effectively induce both humoral and cellular immune responses [33,34]. Because antigenic epitopes interact with lymphocyte surface receptors to stimulate an immune response and thus play a vital role in protein antigenicity, we focused in this study on preparing vaccines fusing antigenic epitopes from Cap2, Cap3, and Cap4 and then evaluating their immunogenicity. Our aim was to lay a scientific foundation for the development of novel subunit vaccines against PCV2, PCV3, and PCV4 infections.

The prokaryotic and baculovirus expression systems are the expression systems most widely used to produce recombinant protein in laboratories [35]. A prokaryotic expression system can rapidly produce a large amount of protein. The utilization of bacteria is facilitated by their simple and cost-effective cell culture methods, along with transcription and translation mechanisms which are widely understood [36]. Baculovirus expression systems are also widely used today; these use strong polyhedrin and P10 promoters to achieve high-level exogenous protein expression without affecting virus infection and replication [37,38]. However, in the present study, the expression levels of rSCap 2-3-4 and rLCap 2-3-4 in the baculovirus expression system were low, and rECap 2-3-4 failed to express, possibly because of the cytotoxic effect of Cap proteins, which may induce host cell lysis. In addition, the N-glycosylation of heterologous proteins produced by insect cells is different from that produced by mammalian cells, affecting final protein expression levels along with stability, biological activity, and immunogenicity [39]. Even when we added a secretory peptide to the N-termini of target sequences to promote secretion from SF9 cells, the recombinant proteins primarily remained intracellular, with limited extracellular presence. Low levels of recombinant proteins pose additional challenges for protein purification, and the high costs associated with eukaryotic expression systems hinder the production of fusion protein vaccines. These findings underscore the need for optimization strategies to enhance the expression efficiency of protein.

Compared with classical vaccines, single-epitope vaccines and multi-epitope vaccines have a unique design concept [40,41,42,43]. Multi-epitope vaccines are composed of various MHC-restricted epitopes that can be recognized by multiple clones of TCRs from various T-cell subsets. These vaccines consist of CTL, TH, and B-cell epitopes, which can simultaneously induce strong humoral and cellular immune responses. Because they are composed of multiple antigenic epitopes of a virus, multi-epitope vaccines can increase the spectra of targeted viral strains [44].

Immune responses play a critical role in resisting viral infections. Therefore, multi-epitope vaccines composed of a series of antigenic epitopes and peptides represent an ideal strategy for preventing and treating infections, which can significantly impact the efficiency of immune protection [44]. Immunization with multi-epitope vaccines has been shown to induce robust cellular and humoral immune responses in mice, providing a certain degree of protection against foot-and-mouth disease virus [42]. In this study, immune response evaluation at 35 d post-immunization revealed that the multi-epitope vaccines group had significantly higher levels of specific antibodies, splenic lymphocyte proliferation, neutralizing antibodies, and levels of cytokines IL-4 and IFN-γ compared with the control group. It is worth noting that immune response in the pSCap 2-3-4 group was higher than in all other immune groups. Previous studies have demonstrated that the VAL-44 multi-epitope vaccine, which contains multiple core protein epitopes of hepatitis C virus as well as CD4^+^ and CD8^+^ T-cell epitopes, induced a strong cellular immune response compared with other groups [45]. For application in mouse models of infection, varying degrees of pathological changes have also been observed in both the lungs and lymph nodes of mice infected with PCV2 [46,47]. In another study, it was demonstrated that the PCV2 DNA vaccine expressing gC1qR binding site mutant Cap induced stronger humoral and cellular immune responses than the PCV2 DNA vaccine expressing wild-type Cap2, and it protected mice from PCV2 infection and lung damage [48]. The recombinant baculoviruses expressing the PCV2 Rep-Cap fusion protein were also shown to produce higher PCV2-specific neutralizing antibody titers and enhanced the protective efficacy in mice [49]. In this study, the absence of an effective PCV4 strain imposed certain limitations, preventing us from assessing the vaccine’s efficacy against PCV4. Therefore, PCV2 and PCV3 were used to infect mice instead. Compared with the control group, the viral load in the lungs of pSCap 2-3-4 group mice was significantly reduced. Considering pathological changes in the lungs of pSCap2-3-4 group mice, we found no obvious bleeding, proliferation, or inflammatory cell infiltration, indicating a protective effect against PCV2 and PCV3 infections. Our finding that the pSCap2-3-4 group exhibited better immunization than other groups might be explained by the high expression and better purification in the *E. coli* expression system. In previous studies, it has been shown that the 169-180 aa region of PCV2 Cap is a decoy epitope, and antibodies against this region do not prevent PCV2 infection [50]. In this study, the viral load in the pLCap2-3-4 group, including the PCV2 Cap 169-180 aa region, was significantly higher than in the pSCap2-3-4 group after PCV2 infection. In addition, the reason for the poor immunoprotection provided by the other two *E. coli* system-expressed proteins could not allow for the exposure of effective antigenic epitopes due to longer epitope junctions resulting in protein folding during expression. However, the low concentration and poor purification of proteins expressed by the baculovirus expression system also resulted in poor immunoprotection. In this study, we used bioinformatics methods to predict B-cell epitopes on the PCV Cap, which not only reduced the length of indicated protein required for efficient expression but also minimized the vaccination dose. A multi-epitope vaccine targeting porcine reproductive and respiratory syndrome virus and Mycoplasma hyopneumoniae was previously reported to exhibit optimal antigenicity and immunogenicity [51]. Our results are consistent with previous findings indicating the protective efficacy of these multi-epitope vaccines. In future studies, we will explore the protective effect of pSCap 2-3-4 vaccine in pigs. This provides a new approach for the development of combined vaccines targeting multiple swine pathogens.

## 5. Conclusions

In conclusion, we found in this study that a multi-epitope vaccine comprising Cap2, Cap3, and Cap4 epitopes effectively prevents PCV2 and PCV3 infection in mice by enhancing both cellular and humoral immune responses. These findings provide valuable insights into potential strategies for developing multi-epitope vaccines against PCV.

## Figures and Tables

**Table 1:** Combinations of Cap antigen epitopes.

	Cap2	Cap3	Cap4
SCap 2-3-4	69-82, 90-104, 121-131, 159-173, 220-234 aa	51-64, 94-107, 120-134 aa	106-178 aa
LCap 2-3-4	65-93, 117-131, 169-183, 175-195, 224-233 aa	42-62, 94-105, 118-139, 147-166, 190-204 aa	106-122, 125-178 aa
ECap 2-3-4	42-234 aa	37-214 aa	38-228 aa

## Data Availability

The data that support the findings of this study are available from the corresponding author upon reasonable request.

## Supplementary Materials

The following supporting information can be downloaded at https://www.mdpi.com/article/10.3390/v16121964/s1, Table S1: Analysis of physical and chemical properties of Cap protein; Figure S1: Prediction of signal peptides and transmembrane domains. (A,C,E) Prediction of signal peptides in Cap2, Cap3, and Cap4. (B,D,F) Prediction of transmembrane domains in Cap2, Cap3, and Cap4; Figure S2: Prediction of secondary structures for Cap2, Cap3, and Cap4. (A) Prediction of secondary structures for Cap2. (B) Prediction of secondary structures for Cap3. (C) Prediction of secondary structures for Cap4. (a) Prediction of protein α-helix structures; (b) Prediction of protein hydrophobicity and hydrophilicity; (c) Prediction of protein flexible regions; (d) Protein antigenic epitope prediction; (e) Prediction of protein surface accessibility; Figure S3: Prediction results of B-cell epitopes for Cap2, Cap3, and Cap4. (A) BCPred prediction results. (B) FBCPred prediction results. (C) AAP prediction results. (D) IEDB prediction results; Figure S4: All nucleotide sequences of pSCap 2-3-4, pLCap 2-3-4, pECap 2-3-4, rSCap 2-3-4, rLCap 2-3-4, and rECap 2-3-4 in different plasmids. Sequences labeled in red indicate linkers connecting predicted antigen epitopes or Cap proteins of PCV2, PCV3, and PCV4. Sequences labeled in purple indicate secreted peptide sequences inserted into eukaryotic vectors. (A) The sequence of pSCap 2-3-4. (B) The sequence of pLCap 2-3-4. (C) The sequence of pECap 2-3-4. (D) The sequence of rSCap 2-3-4. (E) The sequence of rLCap 2-3-4. (F) The sequence of rECap 2-3-4.
