# Supplementary material for: A Subunit Vaccine Harboring the Fusion Capsid Proteins of Porcine Circovirus Types 2, 3, and 4 Induces Protective Immune Responses in a Mouse Model
Source: Viruses. 2024 Dec 23;16(12):1964. doi: 10.3390/v16121964 (PMC11728783; doi:10.3390/v16121964)
Supplement: Supplementary file 1 [file viruses-16-01964-s001.zip › viruses-3239871-supplementary.pdf]

Supplementary Material

A Porcine Circovirus Subunit Vaccine Expressing Fusion Capsid Proteins of Porcine Circovirus Genotypes 2, 3 and 4 Elicits Immunogenicity and Protective Efficacy in Mice

Qikai Wang [1,†], Ran Zhang [1,†], Yue Wang [1], Ying Wang [1], Libin Liang [1], Haili Ma [1], Haidong Wang [1], Longlong Si [2,3,]* and Xingchen Wu [1,4,]*

*** Correspondence:** ll.si@siat.ac.cn (L.S.); wuxingchen@sxau.edu.cn (X.W.)

1. Supplementary table

Table S1 Analysis of physical and chemical properties of Cap protein

	**Cap2**	**Cap3**	**Cap4**
**Amino acid（Pcs）**	234	214	228
**Molecular formula**	$C_{1262}H_{1938}N_{378}O_{337}S_5$	$C_{1158}H_{1784}N_{334}O_{303}S_6$	$C_{1227}H_{1868}N_{378}O_{330}S_3$
**Protein Molecular Weight（KDa）**	27.958	25.425	27.291
**Isoelectric Point（PI）**	10.75	10.84	5.63
**Number of amino acid residues (Pcs)**	56	51	53
**Number of positively charged residues（Arg+Lys）**	16	12	14
**Number of negatively charged residues（Asp+Glu）**	40	39	39
**Average hydrophilic coefficient（GRAVY）**	−0.826	−0.752	−0.879

2. Supplementary figures

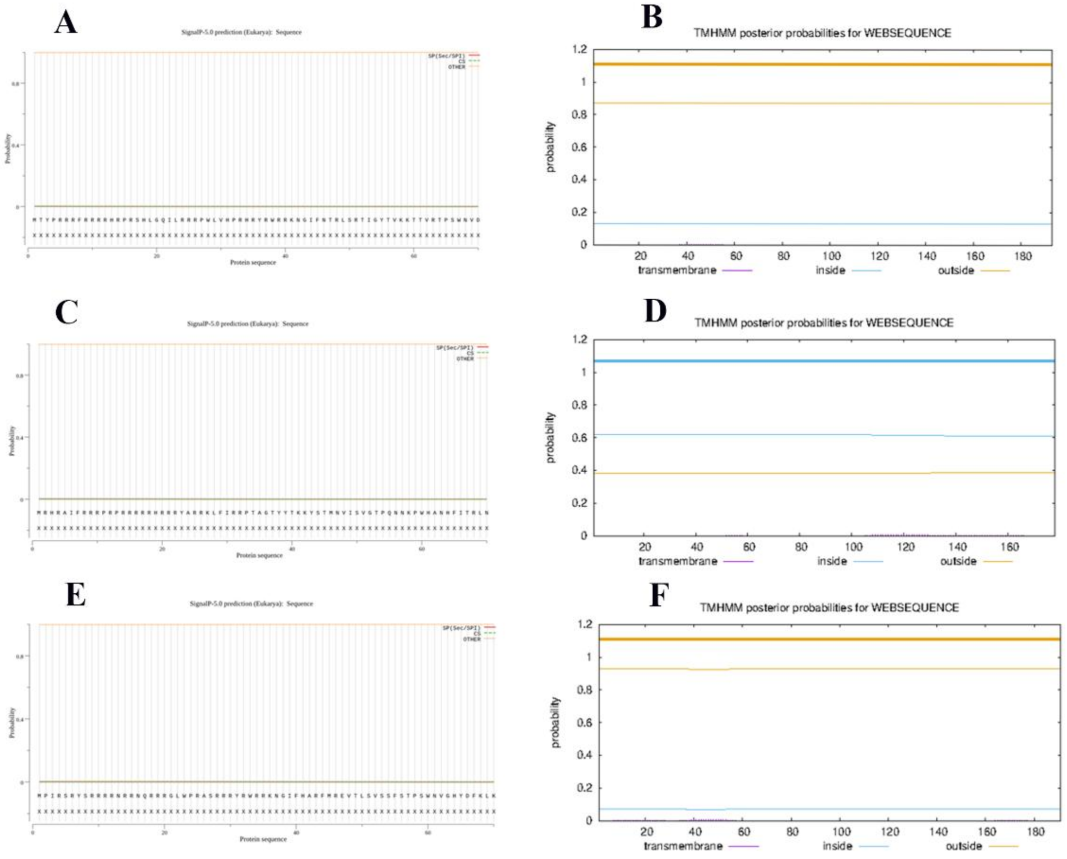

Fig S1. Prediction of signal peptides and transmembrane domains. (A, C, E) Prediction of signal peptides in Cap2, Cap3, and Cap4. **(B, D, F)** Prediction of transmembrane domains in Cap2, Cap3, and Cap4.

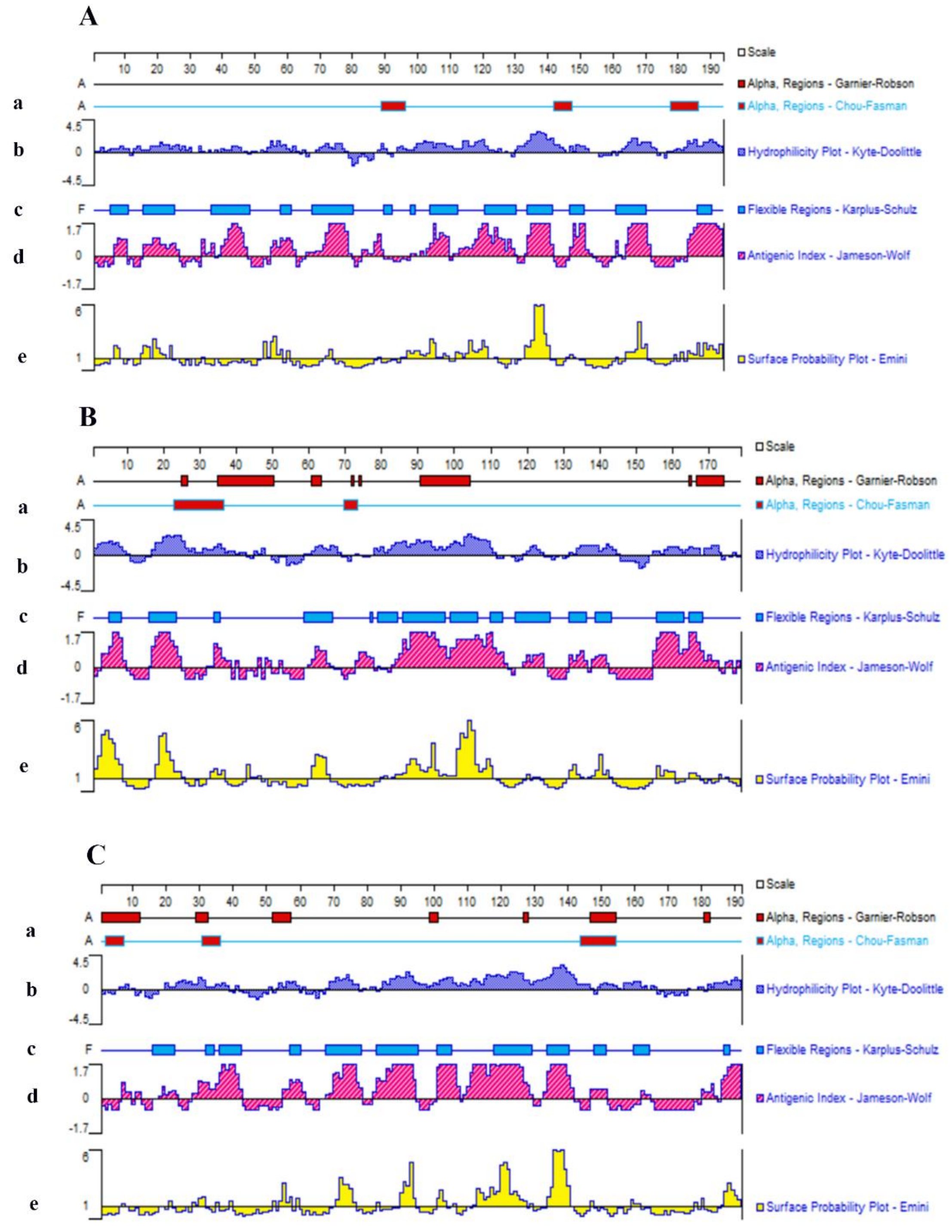

Fig S2. Prediction of secondary structures for Cap2, Cap3, and Cap4. (A) Prediction of secondary structures for Cap2. **(B)** Prediction of secondary structures for Cap3. **(C)** Prediction of secondary structures for Cap4. **a** Prediction of protein α-helix structures; **b** Prediction of protein hydrophobicity and hydrophilicity; **c** Prediction of protein flexible regions; **d** Protein antigenic epitope prediction; **e** Prediction of protein surface accessibility.

A

Cap2

Position	Epitope	Score
1	MTYPRRRFRRRRHRPRSHLG	1
73	RFNINDFLPPGGGSNPLTVP	1
162	TPKPVLDGTIDYFQPNNKRN	1
23	LRRRPWLVHPRHRYRWRRKN	0.998
215	ITMYVQFREFNLKDPPLNPK	0.965
98	IRKVKVEFWPCSPITQGDRG	0.957
137	TYDPYVNYSSRHTITQPFSY	0.941

Cap3

Position	Epitope	Score
13	PRRRRRHRRRYARRKLFIRR	1
114	AWTTNTWLQDDPYAESSTRK	1
45	TMNVISVGTPQNNKPWHANH	1
164	FFFSRPTPWLNTYDPTVQWG	1
195	TGMTDFYGTKEVWIRYKSVL	0.938
67	TRLNEWETAISFEYYKILKM	0.76

Cap4

Position	Epitope	Score
6	RYSRRRRNRRNQRRRGLWPR	1
148	ARHSRYFTPKPQDPSGETHT	1
51	SVSSFSTPSWNVGHYDFKLK	1
28	RRRYRWRRKNGIFHARFMRE	0.987
169	HFQPNNKRNQWWISMADQDL	0.971
194	QYSIQNSNFVQVWTVRFTLY	0.848

B

Position	Epitope	Score
1	MTYPRRRFRRRRHRPRSHLG	1
73	RFNINDFLPPGGGSNPLTVP	1
162	TPKPVLDGTIDYFQPNNKRN	1
23	LRRRPWLVHPRHRYRWRRKN	0.998
215	ITMYVQFREFNLKDPPLNPK	0.965
98	IRKVKVEFWPCSPITQGDRG	0.957
137	TYDPYVNYSSRHTITQPFSY	0.941

Position	Epitope	Score
13	PRRRRRHRRRYARRKLFIRR	1
114	AWTTNTWLQDDPYAESSTRK	1
45	TMNVISVGTPQNNKPWHANH	1
164	FFFSRPTPWLNTYDPTVQWG	1
195	TGMTDFYGTKEVWIRYKSVL	0.938
67	TRLNEWETAISFEYYKILKM	0.76

Position	Epitope	Score
6	RYSRRRRNRRNQRRRGLWPR	1
148	ARHSRYFTPKPQDPSGETHT	1
51	SVSSFSTPSWNVGHYDFKLK	1
28	RRRYRWRRKNGIFHARFMRE	0.987
169	HFQPNNKRNQWWISMADQDL	0.971
194	QYSIQNSNFVQVWTVRFTLY	0.848

C

Position	Epitope	Score
175	QPNNKRNQLWLRLQTTGNVD	1
81	PPGGGSNPLTVPFEYYRIRK	1
148	HTITQPFSYHSRYFTPKPVL	1
126	DDNFVTKANALTYDPYVNYS	0.646

Position	Epitope	Score
9[illegible]	[illegible]	1
190	YVPEKTGMTDFYGTKEVWIR	1
23	YARRKLFIRRPTAGTYYTKK	1
113	GAWTTNTWLQDDPYAESSTR	1
168	RPTPWLNTYDPTVQWGALLW	0.993
66	ITRLNEWETAISFEYYKILK	0.961
145	FTPKPILAGTTSAHPGQSLF	0.131

Position	Epitope	Score
73	IPKGPGTIVNLYSLPFAYYR	1
106	INSNRTYSSTAIQLDGDYVG	1
209	RFTLYVQFREFDLVNYPKQG	0.998
155	TPKPQDPSGETHTLHFQPNN	0.995
11	RRNRRNQRRRGLWPRASRRR	0.137

D

No.	Start	End	Peptide	Length
1	5	20	RRRFRRRRHRPRSHLG	16
2	24	25	RR	2
3	27	38	PWLVHPRHRYRW	12
4	58	68	KKTTVRTPSWN	11
5	80	93	LPPGGGSNPLTVPF	14
6	109	118	SPITQGDRGV	10
7	129	158	FVTKANALTYDPYVNYSSRHTITQPFSYHS	30
8	170	181	TIDYFQPNNKRN	12
9	204	210	NSIYDQD	7
10	224	231	FNLKDPPL	8

No.	Start	End	Peptide	Length
1	5	26	AIFRRRPRFRRRRRHRRRYARR	22
2	45	61	TMNVISVGTPQNNKPWH	17
3	71	81	EWETAISFEYY	11
4	94	105	ISPAQQTKTMFG	12
5	113	160	GAWTTNTWLQDDPYAESSTRKVMTSKKKHSRYFTPKPILAGTTSAHPG	48
6	169	178	PTPWLNTYDP	10
7	189	204	IYVPEKTGMTDFYGTK	16

No.	Start	End	Peptide	Length
1	5	35	SRYSRRRRNRRNQRRRGLWPRASRRRTRWRR	31
2	52	63	VSSFSTPSWNVG	12
3	72	88	FIPKGPGTIVNLYSLPF	17
4	104	112	NGINSNRTY	9
5	122	178	DYVGRGKNQTYDVLANHSSSRHGFTNIARRSRYFTPKPQDPSGETHTLHFQPNNKRNQ	57
6	185	185	D	1
7	199	205	NSNFVQV	7
8	219	225	FDLVNYP	7

Fig S3. Prediction results of B-cell epitopes for Cap2, Cap3, and Cap4. **(A)** BCPred prediction results. **(B)** FBCPred prediction results. **(C)** AAP prediction results. **(D)** IEDB prediction results.

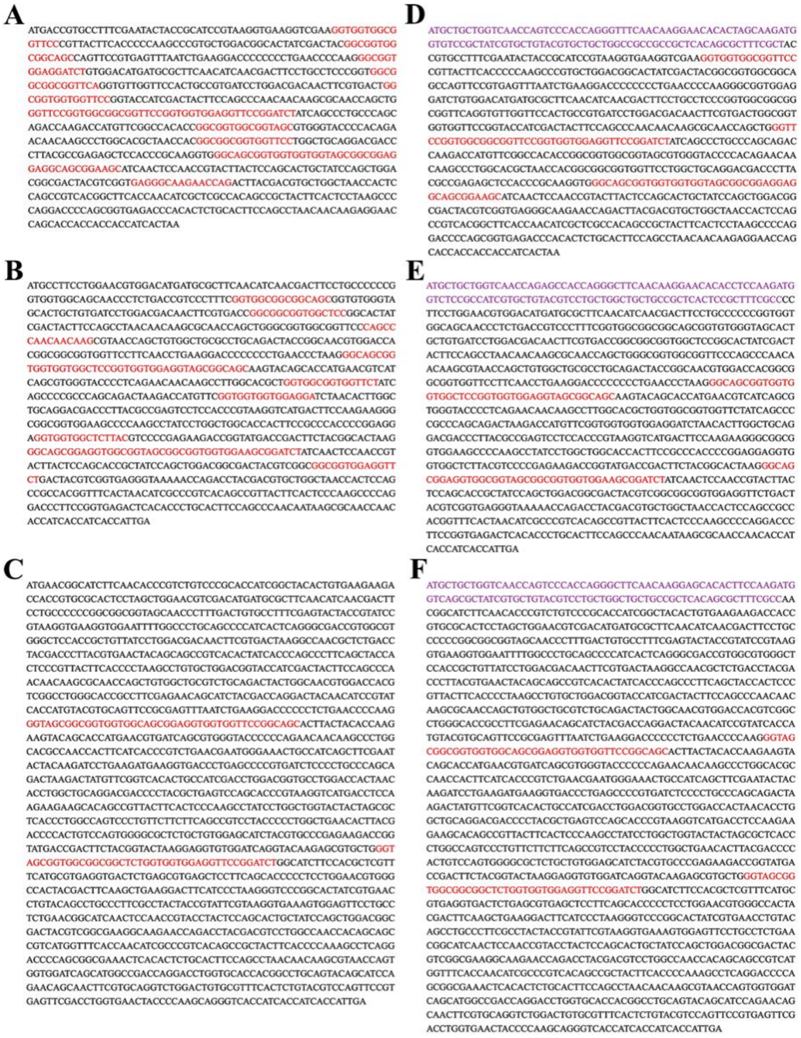

Fig S4. All nucleotide sequences of pSCap 2–3–4, pLCap 2–3–4, pECap 2–3–4, rSCap 2–3–4, rLCap 2–3–4, and rECap 2–3–4 in different plasmids. Sequences labeled in red indicate linkers connecting predicted antigen epitopes or Cap proteins of PCV2, PCV3, and PCV4. Sequences labeled in purple indicate secreted peptide sequences inserted into eukaryotic vectors. **(A)** The sequence of pSCap 2–3–4. **(B)** The sequence of pLCap 2–3–4. **(C)** The sequence of pECap 2–3–4. **(D)** The sequence of rSCap 2–3–4. **(E)** The sequence of rLCap 2–3–4. **(F)** The sequence of rECap 2–3–4.
